# Percutaneous Coronary Intervention after Fibrinolysis for ST-Segment Elevation Myocardial Infarction Patients: An Updated Systematic Review and Meta-Analysis

**DOI:** 10.1371/journal.pone.0141855

**Published:** 2015-11-02

**Authors:** Feng Liu, Qinglong Guo, Guoqiang Xie, Han Zhang, Yaxi Wu, Lixia Yang

**Affiliations:** 1 Department of Postgraduate, Third Military Medical University, Chongqing, China; 2 Department of Postgraduate, Second Military Medical University, Shanghai, China; 3 Department of Cardiology, Kunming General Hospital of Chengdu Military Area, Yunnan, China; 4 Department of Postgraduate, Kunming Medical University, Yunnan, China; Azienda Ospedaliero-Universitaria Careggi, ITALY

## Abstract

**Background:**

Percutaneous coronary intervention (PCI), fibrinolysis and the combination of both methods are current therapeutic options for patients with ST-segment elevation myocardial infarction (STEMI).

**Methods:**

We searched PubMed, EMBASE, Google scholar and Cochrane Controlled Trials Register for randomized controlled trials (RCTs) evaluating the efficacy and safety of PCI after fibrinolysis within 24 hours, which was compared with primary PCI alone and ischemia-guided or delayed PCI. Meta-analysis was conducted using Review Manager 5.30 following the methods described by the Cochrane library.

**Results:**

A total of 16 studies including 10,034 patients were enrolled. As compared with primary PCI alone group, the short-term mortality (5.8% vs 4.5%, RR 1.29, 95% confidence interval [CI] 1.00–1.65) and re-infarction rate (4.1% vs 2.7%, RR 1.46, 95%CI 1.05–2.03) were higher in the immediate PCI group (median/mean time ≤ 2 h after fibrinolysis). However, the short-term mortality and re-infarction rate showed no statistically significant differences in the early PCI group (2–24 hours after fibrinolysis). The rate of major bleeding events was higher both in the immediate PCI (6.3% vs 4.4%, RR 1.43, 95%CI 1.11–1.85) and the early PCI group (6.4% vs 4.4%, RR 1.46, 95%CI 1.03–2.06) as compared with primary PCI alone group. As compared with ischemia-guided or delayed PCI, early PCI was associated with significantly reduced re-infarction (2.4% vs 4.0%, RR 0.61, 95%CI 0.41–0.92) and recurrent ischemia (1.5% vs 5.3%, RR 0.29, 95%CI 0.12–0.70) at short-term. And the reduced re-infarction rate was also observed at long-term.

**Conclusions:**

Early PCI after fibrinolysis, with a relatively broader time for PCI preparation, can bring the similar effects with primary PCI alone and is better than ischemia-guided or delayed PCI in STEMI patients with symptom onset < 12 h who cannot receive timely PCI. However, immediate PCI after fibrinolysis is detrimental.

## Introduction

Primary PCI performed in a timely fashion [< 90 min of first medical contact (FMC)-device time in PCI-capable hospital, and < 120 min of FMC-device time in non-PCI-capable hospital] is the preferred strategy for the treatment of ST-segment elevation myocardial infarction (STEMI) patients with symptom onset < 12 h[1,2]. Nevertheless, the time interval is very strict in the real world setting, especially for patients first admitted to a non-PCI-capable hospital[3,4]. Therefore, PCI after fibrinolysis, which would improve the reperfusion of the affected myocardial muscle earlier, might be suitable for a large portion of STEMI patients.

Clinical trials and several meta-analyses[5–8] were conducted to explore the status and significance of PCI after fibrinolysis using combined endpoints. However, the more detailed and long-term data on specific efficacy endpoints (such as death and re-infarction) and safety endpoints (such as major bleeding) were unclear. Several years after the last detailed meta-analysis, several new studies and long-term outcomes of early studies were published. This study is an updated meta-analysis assessing the efficacy and safety of PCI after fibrinolysis (immediate or early PCI) comparing with primary PCI alone and with ischemia-guided or delayed PCI after fibrinolysis in STEMI patients with symptom onset < 12 h who can not receive timely PCI.

## Methods

### Search strategy

We conducted a systematic search for RCTs published before January 2015 on Pubmed, Embase, Cochrane library and Google scholar using those key words “fibrinolysis/fibrinolytic agent/thrombolysis/thrombolytic agent” and “angiography/angioplasty/percutaneous coronary intervention/PCI” and “ST segment elevation myocardial infarction/ST-segment elevation myocardial infarction/STEMI/acute myocardial infarction/AMI” in title or abstract terms. Bibliography citations were hand-searched from previous meta-analyses and related review articles. We also searched references from the annual scientific session of the American Heart Association (AHA), American College of Cardiology (ACC) and European Society of Cardiology (ESC) meetings.

### Study inclusion

Studies were included by two authors (F. Liu and Q. Guo) independently according to the following criteria. The including criteria were: 1) STEMI patients with symptom onset < 12 h; 2) PCI within 24 hours after fibrinolysis versus primary PCI alone and/or versus ischemia-guided or delayed PCI. The exclusion criteria were: 1) non RCT; 2) without clinical endpoints; 3) conducted in “balloon era” when only balloon was used to open the infarct-related coronary artery.

### Risk of bias assessment

Two authors (Q. Guo and G. Xie) assessed the quality and risk of bias of the included studies independently. The GRADE[9] system on Review Manager was applied to evaluate the quality of the trials, including the methods used to generate the allocation sequence and to conceal allocation to the treatment groups, the blinding of participants and personnel, and the blinding of outcome assessment, the description of incomplete data and the risk of selective outcome reporting. Publication bias was assessed by funnel plots of each comparison.

### Definitions

Two authors (F. Liu and G. Xie) independently reviewed all full-texts and extracted the data of the included studies. In cases of incomplete or unclear data, the reviewers would reach an agreement through discussion. Immediate PCI was defined as PCI conducted with a median/mean time ≤ 2 h after fibrinolysis. And early PCI was defined as PCI with a median/mean time 2–24 h after fibrinolysis. We chose the specific clinical outcomes as the efficacy endpoints, consisting of mortality, re-infarction, recurrent ischemia during hospitalization or at 1, 3, 12 months, and longer. Short-term outcomes were defined as those within 1 month, 3 months and during hospitalization, while long-term ones were defined as those occurred one year or longer. Major bleeding within 30 days was evaluated as the safety endpoint. As the definitions of major bleeding varied among different studies, we defined major bleeding events as any bleeding complications causing death, intracranial hemorrhage, substantial hemodynamic compromise requiring treatment, need for transfusion, a decrease in hemoglobin of more than 5 mg/dl or in hematocrit of more than 15%, which would incorporate the definitions of major Thrombolysis in Myocardial Infarction (TIMI) bleedings[10] and the severe or moderate Global Utilization of Streptokinase and Tissue Plasminogen Activator for Occluded Coronary Arteries (GUSTO) bleedings[11].

### Statistical analysis

The Cochrane Collaboration Review Manager (Rev Man, Version 5.30) was used to conduct the data pooling and statistical analysis. Risk ratio (RR) and 95% confidence interval (CI) were used as summary statistics. The heterogeneity between studies was analyzed using *I* ^*2*^. Pooled statistics were calculated using the Mantel-Haenszel random-effects model. A two-sided p value < 0.05 was considered statistically significant. This meta-analysis was performed according to the Preferred Reporting Items for Systematic Reviews and Meta-Analyses (PRISMA) statement.

## Results

### Study selection

**Fig 1:**
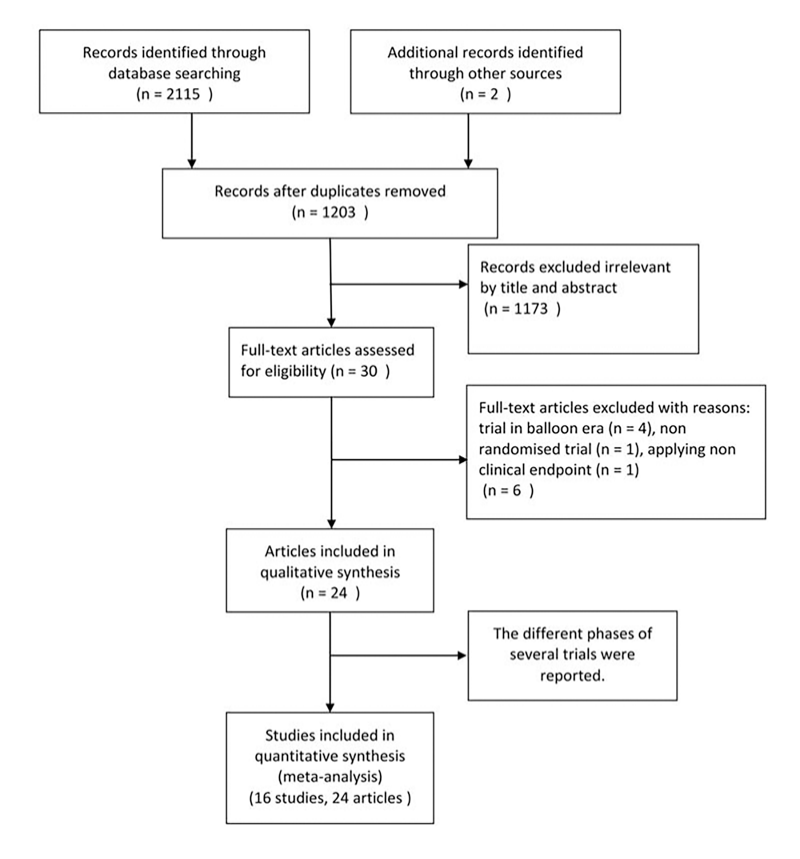
The flow chart of literature inclusion. (Flow Diagram).

There were 1,203 records obtained by database searching and 30 full-text articles were assessed for eligibility. We excluded 4 trials[12–15] conducted in “balloon era”, 1 non randomized trial[16] and 1 applying non clinical endpoints[17]. Finally, a total of 16 studies including 24 articles[18–41] incorporating 10,034 patients were included in our meta-analysis. There were 7 studies[19,21,22,24–27] referring to immediate or early PCI after fibrinolysis versus primary PCI alone. The comparison of immediate or early PCI versus ischemia–guided or delayed PCI after fibrinolysis was explored in 7 trials[28,30,33–36,37]. Both comparisons were described in the other 2 studies[39,41]. The procedure of searching and including studies was shown on the Flow Diagram (**Fig 1**).

### Quality of included studies

**Fig 2:**
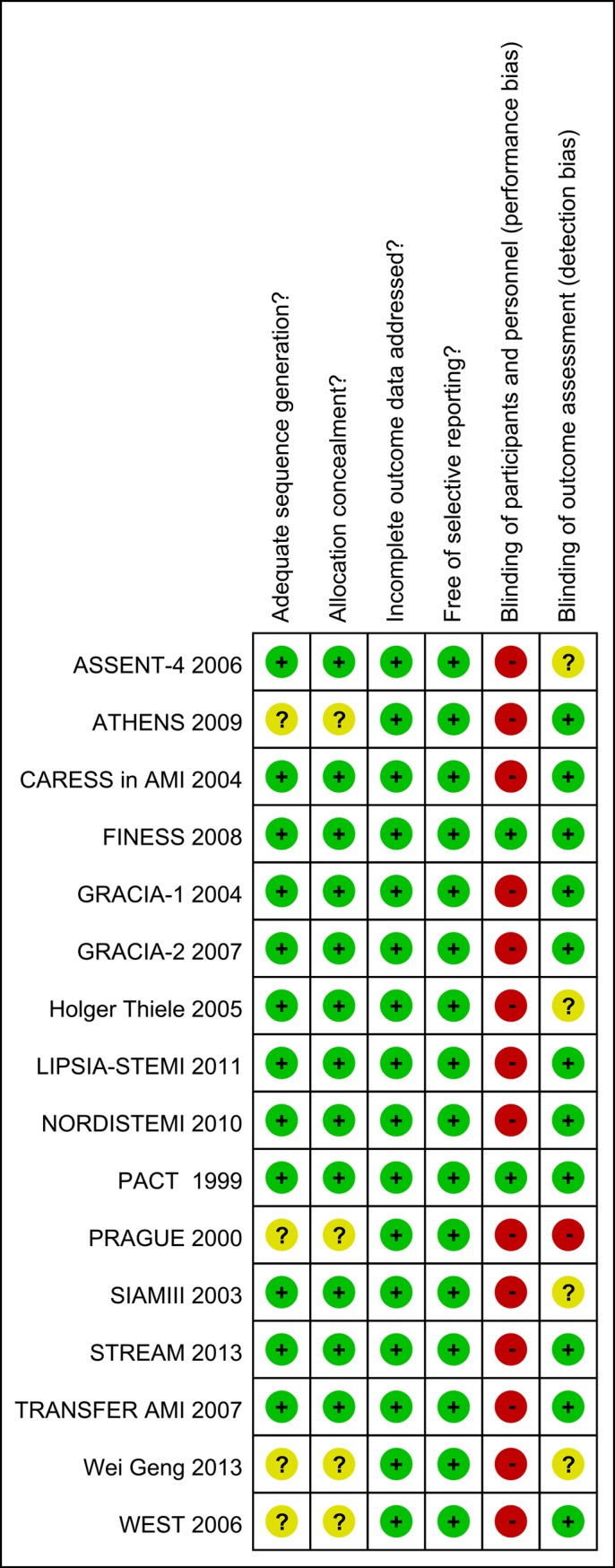
Risk of bias of included studies.

Results of the quality assessment were listed in **Fig 2**. Two trials[24,26] of those RCTs were double blinded. Patients and investigators were not blinded to treatment in the remaining 14 trials. However, blinding of the clinical endpoint assessment was used in 11 studies[19,21,24–28,30,33,36,41]. Therefore, clinical outcomes were less likely influenced by the lack of blinding. Only 4 trials[27,34,40,41] did not describe the methods of sequence generation and allocation concealment. The bias of selective reporting and incomplete data was low in all trials.

### Immediate or early PCI after fibrinolysis versus primary PCI alone

Nine studies[19,21,22,24–27,39,41] (6,862 patients) were identified for the comparison of immediate or early PCI after fibrinolysis versus primary PCI alone. The basic characteristics were listed in **Table 1**. The Plasminogen-activator Angioplasty Compatibility Trial (PACT)[26] study excluded patients >75 years and the Facilitated Intervention with Enhanced Reperfusion Speed to Stop Events (FINESS) study[23,24] excluded patients <60 years. The remaining trials applied the similar inclusion and exclusion criteria (**Table 1**) There were some differences in the use of fibrinolytic agents: standard weight-adjusted tenecteplase in 5 trials[19,21,22,25,41], reteplase[24] in 1 trial, streptokinase[39] in 1 trial, rt-PA[26] in 1 trial and 1/4 weight-adjusted tenecteplase[27] in 1 trial. Clopidogrel was used in 7 trials[19,21,22,24,25,27,41]. Glycoprotein IIb/IIIa receptor antagonists were used to the discretion of the investigators in 7 trials[19,21,22,24,25,27,41]. Asprin and heparin were used in all trials. PCI was performed with a median/mean time within 2 h after fibrinolysis in 6 trials[22,24–27,39]. In 3 studies[19,21,41], PCI was performed within 2–24 h after fibrinolysis.

**Table 1:** Characteristics of the included trials. (Immediate or early PCI after fibrinolysis versus primary PCI alone).

*Trials*	*Year*	*Number of patients*	*Age (y)*	*Gender (male %)*	*Characteristics of patients*		*From symptom onset to fibrinolysis (h)* ^*c*^	*From fibrinolysis to angiography (h)* ^*c*^	*Type and dose of fibrinolytic drugs*
		*A/B* ^*a*^	*A/B*	*A/B*	Major Inclusion criteria	Major exclusion criteria	*B*	*B*	*B*
***Immediate PCI***									
PACT^26^	1999	304/302	58.4/57.6	78.3/79.1	age> 18 y, symptom onset <6 h	age≥75 y, contraindications to lysis	2.2	0.8	rt-PA
PRAGUE^39,40^	2000/2003	101/100	61/62	72/73	symptom onset <6 h	contraindications to lysis	2.1	1.6	Streptokinase
ASSENT-4^22^	2006	838/829	60/61	77/77	age> 18 y, symptom onset <6 h	contraindications to lysis	2.6	1.7	weight-adjusted tenecteplase
FINESS^23,24^	2008/2009	806/828	62.5/62.6	73.6/74.3	symptom onset <6 h, expected arrival to catheterization 1–4 h after randomization	low risk patients^**b**^	2.8	1.5	abciximab and reteplase^**d**^
ATHENS^27^	2009	141/143	59/61	89/92	age> 18 y, symptom onset <6 h	expected arrival to catheterization < 30min after randomization, contraindications to lysis	2.3	2.0	1/4 weight-adjusted tenecteplase
LIPSIA-STEMI^25^	2011	81/81	61/63	76/82	symptom onset <3 h	contraindications to lysis and MRI	1.1	1.6	weight-adjusted tenecteplase
***Early PCI***									
WEST^41^	2006	100/104	60/57	82/81	age> 18 y, symptom onset <3 h	contraindications to lysis	2.2	4.5	weight-adjusted tenecteplase
GRACIA-2^21^	2007	108/104	67.5/61	82.4/79.8	symptom onset <12 h	contraindications to lysis, renal failure	3	4.6	weight-adjusted tenecetplase
STREAM^18,19,20^	2010/2013/2014	948/944	59.6/59.7	78.1/79.4	age> 18 y, symptom onset <3 h	expected primary PCI < 60 min from diagnosis, acute pancreatitis or severe hepatic dysfunction, renal insufficiency	1.7	8.1	weight-adjusted tenecteplase

Abbreviations

^**a**^. A = the primary PCI alone group, B = the immediate or early PCI group

^**b**^. low risk, i.e., if they were < 60 years old and had a localized inferior infarction

^**c**^. The description of age and time was median or mean value

^**d**^. Two 5-U boluses separated by 30 minutes for those < 75 years old, one 5-U dose for those ≥ 75 years old

**Fig 3:**
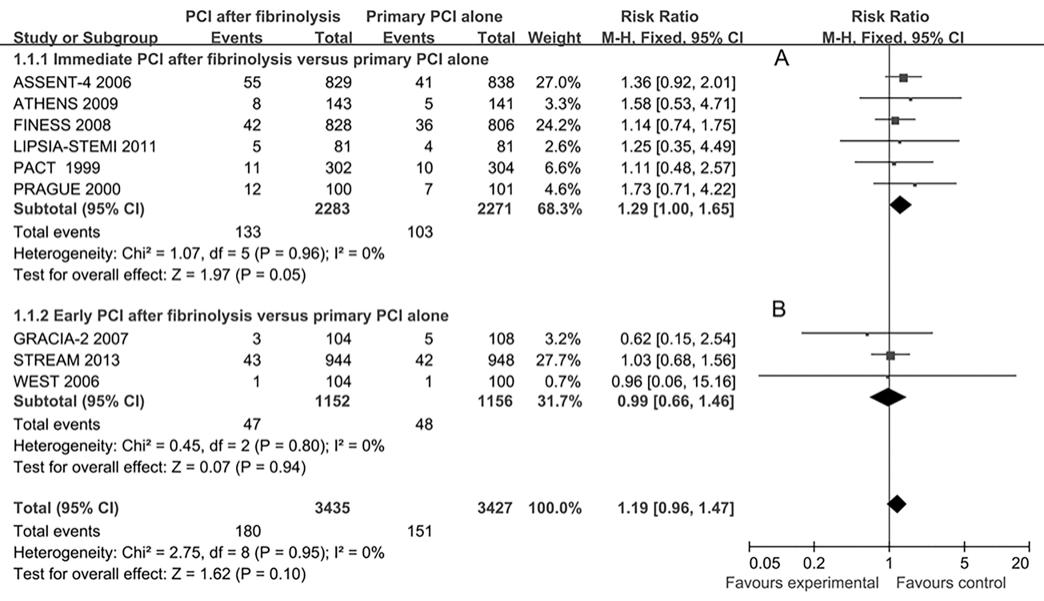
Short-term mortality for immediate or early percutaneous coronary intervention (PCI) after fibrinolysis versus primary PCI alone.

All studies presented short-term mortality and 3 studies[20,24,39] reported long-term mortality. As compared with the primary PCI alone group, there were no statistically significant differences between death rates at short-term (9 studies, RR 1.19, 95%CI 0.96–1.47, **Fig 3**) and at long-term (3 studies, RR 1.01, 95%CI 0.79–1.28) in the immediate or early PCI group. Subgroup analysis showed that the short-term mortality was higher in the immediate PCI group (6 studies, RR 1.29, 95%CI 1.00–1.65, **Fig 3A**), but without statistically significant difference in the early PCI group (3 studies, RR 0.99, 95%CI 0.66–1.46, **Fig 3B**) as compared with primary PCI alone group. Long-term mortality showed no statistically significant differences in the immediate PCI group, the early PCI group and the total effects (3 trials, 6.7% versus 6.7%, RR 1.01, 95% CI 0.79–1.28) as compared with the primary PCI alone group.

**Fig 4:**
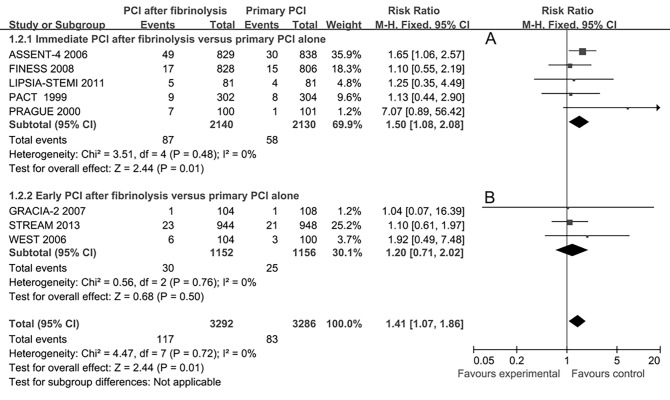
Short-term re-infarction for immediate or early percutaneous coronary intervention (PCI) after fibrinolysis versus primary PCI alone.

More re-infarction events were observed in the immediate or early PCI group as compared with primary PCI alone group (9 studies, RR 1.41, 95%CI 1.07–1.86, **Fig 4**). Subgroup analysis showed that the short-term re-infarction rate was higher in the immediate PCI group (5 studies, RR 1.50, 95%CI 1.08–2.08, **Fig 4A**) and without statistically significant difference in the early PCI group (3 studies, RR 1.20, 95%CI 0.71–2.02, **Fig 4B**) as compared with the primary PCI alone group.

**Fig 5:**
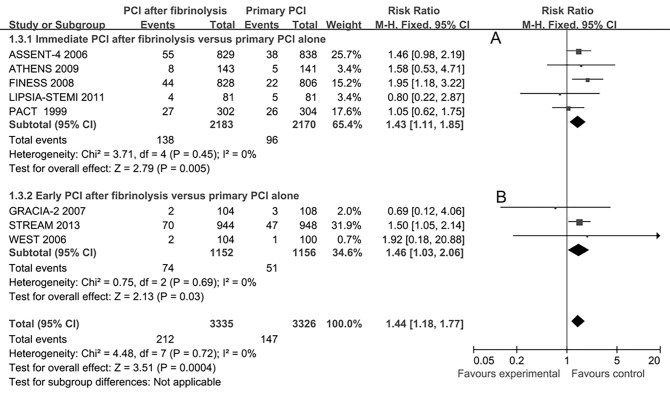
Major bleeding within 30 days for immediate or early percutaneous coronary intervention (PCI) after fibrinolysis versus primary PCI alone.

Major bleeding within 30 days was reported in 8 studies. There were more major bleeding events in the immediate PCI group (5 studies, RR 1.43, 95%CI 1.11–1.85, **Fig 5A**), the early PCI group (3 studies, RR 1.46, 95%CI 1.03–2.06, **Fig 5B**) and the total effects (RR 1.44, 95%CI 1.18–1.77, **Fig 5**) as compared with the primary PCI alone group.

### Immediate or early PCI after fibrinolysis versus ischemia-guided or delayed PCI

Nine studies[28,30,33–36,37,39,41] involving 3,376 patients were included. The basic characteristics of the studies were listed in **Table 2**. The use of fibrinolytic agents was as follows: standard weight-adjusted tenecteplase was used in 3 studies[28,30,41], reteplase in 2 trials[34,37], abciximab and half-dose reteplase in 2 studies[33,35], streptokinase in 1 study[40] and alteplase in 1 study[36]. According to the median/mean time from fibrinolysis to PCI, there were 2 trials[35,39] in the immediate PCI group and 7 trials[28,30,33,34,36,37,41] in the early PCI group.

**Table 2:** Characteristics of the included trials. (Immediate or early PCI after fibrinolysis versus ischemia-guided or delayed PCI).

*Trials*	*Year*	*number of patients*	*Age (y)*	*Gender (male %)*	*characteristics of patients*		*time from symptom onset to fibrinolysis (h)*		*time from fibrinolysis to angiography (h)*		*type and dose of fibrinolytic drugs*	*rate of angiograhy (%)*
		*B/C* ^a^	*B/C*	*B/C*	Major inclusion criteria	Major exclusion criteria	*B*	*C*	*B*	*C*	*B and C*	*B/C*
***Immediate PCI***												
PRAGUE^39,40^	2000/2003	100/99	62/61	73/68	symptom onset <6 h	contraindications to lysis	2.1	2.0	1.6	NA^**b**^	Streptokinase	100/7.1
Holger Thiele et al^35^	2005	82/82	65/60	74/78	symptom onset <6 h	contraindications to lysis	1.5	1.5	1.5	NA	abciximab and 1/2 reteplase	96/91
***Early PCI***												
CARESS in AMI^32,33^	2004/2008	298/300	60.2/59.6	77.9/79.3	symptom onset <12 h	Age>75 y, contraindications for lysis, severe chronic renal or hepatic impairment	2.8	2.7	2.3	3.5	abciximab and 1/2 reteplase	97/35.7
NORDISTEMI^28^	2010	134/132	60/61	80/71	Age 18–75 y, symptom onset <6 h	contraindications to lysis serious renal failure	2.0	2.1	2.7	3 d	weight adjusted dose tenecteplase	99/95
SIAMIII^37,38^	2003/2011	82/81	62.4/63.4	76.8/80.2	age>18 y, symptom onset <12 h	contraindications to lysis, severe renal failure	3.2	3.6	3.5	11.7 d	Reteplase	NA/NA
GRACIA-1^36^	2004	248/251	60/61	87/85	age> 18 y, symptom onset <12 h	contraindications to lysis, renal failure	3.0	3.1	16.6	NA	alteplase	NA/NA
WEST^41^	2006	104/100	57/58	81/75	age> 18 y, symptom onset <3 h	contraindications to lysis	2.2	1.9	4.5	5.0	weight-adjusted tenecteplase	78/58
TRANSFER AMI^29,30,31^	2007/2009/2012	537/522	56/57	79.3/79.9	symptom onset <12 h	contraindications to lysis	1.9	1.9	3.9	22.7	weight-adjusted tenecteplase	98.5/88.7
Wei Geng et al^34^	2013	112/112	59.2/60.3	73.2/76.8	age> 18 y, symptom onset <12 h	Age>75 y, contraindications to lysis	2.7	3.0	10	8 d	reteplase	92/89.3

Abbreviations

^**a**^. B = the immediate or early PCI group, C = the ischemia-guided or delayed PCI group

^**b**^. NA = not applicable

**Fig 6:**
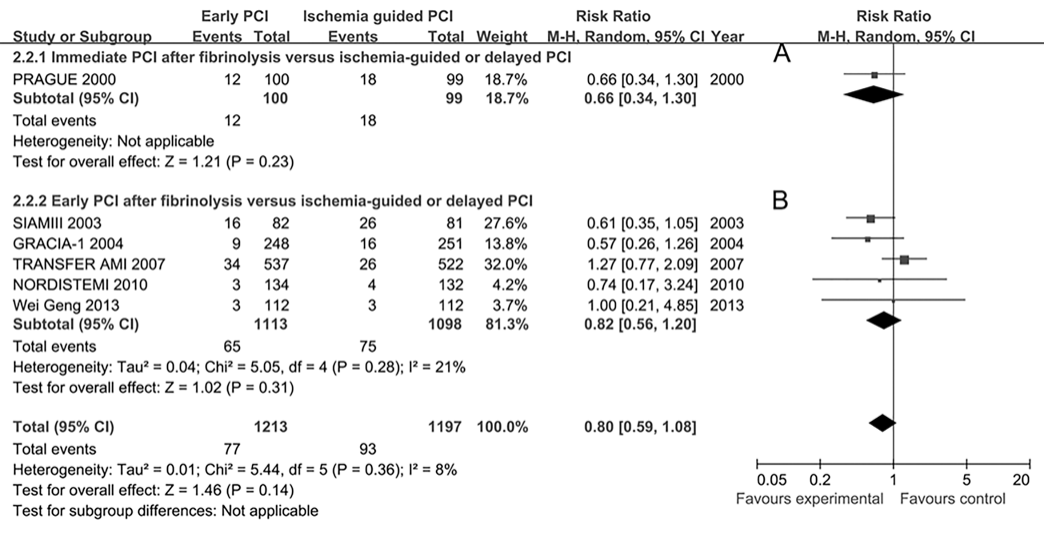
Long-term mortality for immediate or early percutaneous coronary intervention (PCI) after fibrinolysis versus ischemia-guided or delayed PCI.

The short-term mortality was available in all studies and the long-term mortality was available in 6 trials[28,30,34,36,38,40]. The overall death rate in the immediate or early PCI group was similar to that in the ischemia-guided or delayed PCI group both at short-term (62/1697 vs 72/1679, RR 0.86, 95%CI 0.62–1.21) and at long-term (77/1213 vs 93/1197, RR 0.80, 95%CI 0.59–1.08, **Fig 6**). Subgroup analysis showed that there were no statistically significant differences of mortalities in the immediate PCI group (2 studies, 14/182 vs 18/181, RR 0.78, 95%CI 0.40–1.51) and the early PCI group (7 studies, 48/1515 vs 54/1498, RR 0.89, 95%CI, 0.60–1.32) at short-term and at long-term (**Fig 6A** and **Fig 6B**) as compared with the ischemia-guided or delayed PCI group.

**Fig 7:**
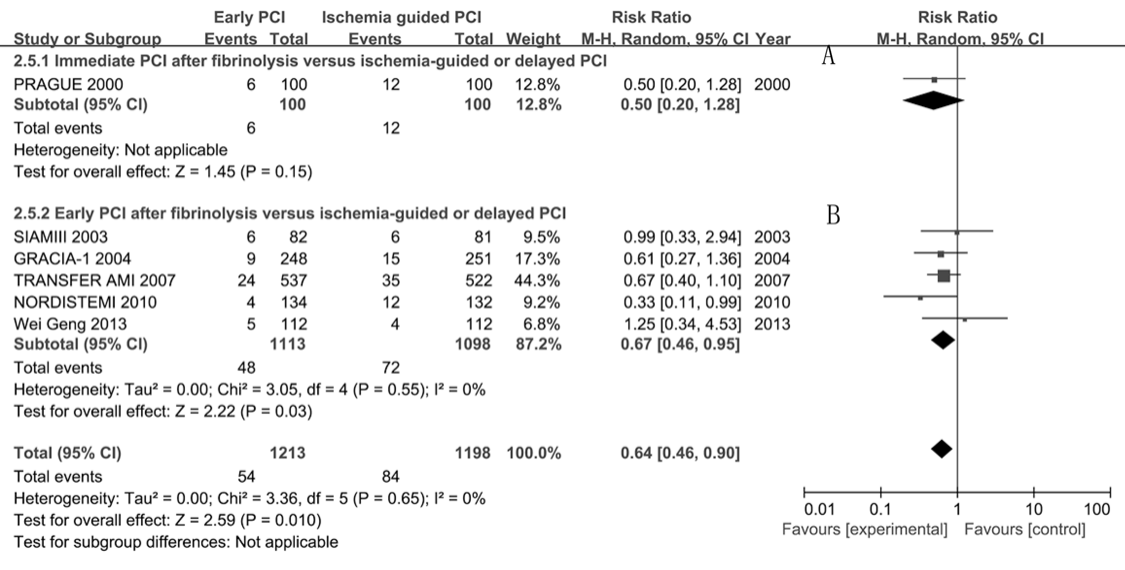
Long-term re-infarction for immediate or early percutaneous coronary intervention (PCI) after fibrinolysis versus ischemia-guided or delayed PCI.

The re-infarction rate was lower in the immediate or early PCI group than that in ischemia-guided or delayed PCI group at short-term (47/1697 vs 77/1679, RR 0.61, 95%CI 0.42–0.87) and long-term (54/1213 vs 84/1198, RR 0.64, 95%CI 0.46–0.90, **Fig 7**). Subgroup analysis showed that compared with ischemia-guided or delayed PCI at short-term, the re-infarction rate was similar in the immediate PCI group (10/182 vs 17/181, RR 0.59, 95%CI 0.28–1.26) but lower in the early PCI group(37/1515 vs 60/1498, RR 0.61, 95%CI 0.41–0.92). Similar effects were observed at long-term (**Fig 7A** and **Fig 7B**).

Recurrent ischemia events were reported in 6 trials[28,30,33,34,37,41], all of which were in early PCI group. Early PCI after fibrinolysis was associated with reduction of recurrent ischemia (1.5% vs 5.3%, RR 0.29, 95%CI 0.12–0.70) as compared with ischemia-guided or delayed PCI at short-term. Only 3 studies reported the long-term recurrent ischemia events but no statistically significant differences were found among different groups (RR 0.60, 95%CI 0.61–1.76).

The major bleeding events within 30 days showed no statistically differences in the comparisons of immediate PCI versus ischemia-guided or delayed PCI (1 study, RR 0.80, 95%CI 0.22–2.87), early PCI versus ischemia-guided or delayed PCI (7 studies, RR 0.94, 95%CI 0.69–1.30), as well as the total effects (8 studies, RR 0.94, 95%CI 0.69–1.27).

### Heterogeneity and stability

The heterogeneity was relatively low in all comparisons because the *I* ^*2*^ was always less than 25%. The funnel plots did not show significant visual asymmetry in the comparisons of mortalities and other endpoints between different groups. Omitting studies one by one showed that the overall meta-analysis estimates would not be turned over by excluding any study in all comparisons, except in one circumstance: if the Strategic Reperfusion Early After Myocardial Infarction (STREAM) trial[19] was omitted, the major bleeding events would be similar between the early PCI group and the primary PCI alone group.

## Discussion

### Summary of results and possible explanations

The present meta-analysis is based on the most updated and detailed data to assess the short-term and long-term efficacy and safety of PCI after fibrinolysis for STEMI patients.

Immediate PCI after fibrinolysis would bring more deaths, re-infarction events and major bleeding events than primary PCI alone. However, it has similar effects as compared with ischemia-guided or delayed PCI both at short-term and long-term. Clinical outcomes have shown the disadvantages of immediate PCI in either full dose fibrinolytic agents, 1/4 dose tenecteplase, or half-dose reteplase plus abciximab as the fibrinolytic strategy. Though in these trials, the percentage of Thrombolysis in Myocardial Infarction (TIMI) grade III flow before PCI was higher than that in primary PCI alone group[27]. However, this didn’t bring benefit to the clinical outcomes. There are probably some reasons that have mitigated the benefit of early better TIMI flow. Firstly, the prothrombotic circumstance accompanied by increased platelet activation and aggregation caused by short application of fibrinolytic agents may have limited the benefit of PCI. Secondly, the use of fibrinolytic agent would soften the fresh thrombus and divide them into small pieces which would lead to hemodynamic instability. Thirdly, differences in time of reperfusion may affect the major myocardial salvage approximately only during first 2 hours after the onset of infarction, and the time interval can hardly be met in many of the included studies which enrolled patients with < 12 h, 6 h or 3 h of symptom onset. Therefore, the time-dependency of PCI-mediated salvage may be considerably attenuated[23]. Fourthly, the increased major bleeding events may also contribute to more deaths.

Early PCI after fibrinolysis, with a relatively broader time for PCI preparation, can bring the similar effects of primary PCI alone and is better than ischemia-guided or delayed PCI. The median/mean time interval from first medical contact or randomization to PCI was longer in the early PCI group as compared with the primary PCI alone group, which was reported in STREAM[19] (492 min vs 77 min), Grupo de Analisis de la Cardiopatia Isquemica Aguda (GRACIA-2)[21] (4.6 h vs 1 h) and Which Early ST-elevation myocardial infarction Therapy (WEST)[41] (324 min vs 127 min) studies. However, the mortality and re-infarction in both groups showed no statistical differences. It means that a broader time for PCI preparation is acquired by pre-transfer fibrinolysis. The TIMI flow before PCI was better than primary PCI alone both in STREAM[18] and GRACIA-2[21] studies, which might have brought better clinical outcomes. In a non-randomized registry trial[4], for the patients with time from onset to call ≤ 12 h and treated with either fibrinolysis or primary PCI, the crude 5-year survival was 88% with the fibrinolysis-based strategy and 83% with intended primary PCI. They concluded that the initial fibrinolytic strategy followed by coronary angiography yielded results that were at least as good as those of primary PCI. Also, a report from the US National Cardiovascular Data Registry[3], showed that patients treated with fibrinolysis requiring inter-hospital transfer had no significant difference on mortality (3.7% vs 3.9%; adjusted odds ratio, 1.13;95% CI,0.94–1.36) as compared with those undergoing primary PCI, but with higher bleeding risk (10.7% vs 9.5%; adjusted odds ratio, 1.17; 95%CI 1.02–1.33).

As compared with ischemia-guided or delayed PCI, the re-infarction and recurrent ischemia events were less in the early PCI group. However, there was no statistically significant difference on mortality. The timely vascular reconstruction and higher PCI rate in the early PCI group might be associated with the similarity of mortalities. High rescue PCI rate in many studies might have lowered the mortality in the ischemia-guided or delayed PCI group. The PCI rate was higher than 80% in ischemia-guided or delayed PCI group in 4 trials[28,30,34,35], 30%-60% in 2 trials[33,41], 7.1% in 1 study[39], but were not mentioned in 2 trials[36,37]. In the practical conditions, fibrinolysis alone could improve the reperfusion of myocardial muscle but rescue PCI was necessary if fibrinolysis failed to open the infarct-related artery. So this analysis excluded the study which compared immediate or early PCI with thrombolysis alone[42].

Major bleeding events occurred more frequently in the immediate or early PCI after fibrinolysis group as compared with primary PCI alone group. In the early PCI group, it was mainly influenced by STREAM[19], in which the major bleeding events were more in the early PCI group and the tenecteplase dose was halved in patients aged ≥75 years at one fifth of the planned enrollment because of an excess of intracranial hemorrhage. After the amendment, no more intracranial hemorrhage events were observed in the elderly group in this study. If this study was omitted, the major bleeding events would become similar to the primary PCI alone group. As compared with ischemia-guided or delayed PCI, there was no difference in major bleeding events. That means the procedure of fibrinolysis might lead to higher major bleeding rate, no matter followed by immediate, early or delayed PCI. Therefore, for patients with an age ≥ 75 years, a reduced dose of fibrinolytic agents is suitable.

### Limitations of this review

There were several differences between included studies, which may induce the heterogeneity. Patients >75 years were excluded in 4 studies[26,28,33,34]. However, the low risk patients were excluded in the FINESS study. Weight-adjusted tenecteplase, alteplase, reteplase and streptokinase have different effects in the fibrinolytic treatment of STEMI patients[1]. Also the reduced doses of the fibrinolytic agents in 3 trials[27,33,35] may cause different effects. Glycoprotein IIb/IIIa receptor antagonists were used to the discretion of the investigators in PCI patients in most trials. Hence, the use of them varied in different groups among different studies. And the follow-up time varied from during hospitalization[27] to 7.9 years[38]. So we divided the follow-up time interval to short-term and long-term to reduce the heterogeneity. The time from symptom onset to fibrinolysis or first medical contact was also different. The time interval from fibrinolysis to PCI varied from 0.8 h[26] to 16.6 h[36]. So we conducted subgroup analyses (immediate PCI or early PCI after fibrinolysis) according to the time interval. However, only median/mean time interval was available in the published data and only 2 sections divided, which would not be sufficient to evaluate the more specific relationship between time interval and clinical outcomes. Future studies should be conducted to reach more specific results.

## Conclusions

This meta-analysis strengthened the evidence that fibrinolysis followed by early PCI is the optimal strategy for STEMI patients with symptom onset < 12 h who can not be transferred to undergo PCI within 120 minutes.

## Supporting Information

S1 PRISMA Checklist PRISMA 2009 Checklist. (DOC)
