# Correction: Percutaneous Coronary Intervention after Fibrinolysis for ST-Segment Elevation Myocardial Infarction Patients: An Updated Systematic Review and Meta-Analysis

**DOI:** 10.1371/journal.pone.0146207

**Published:** 2015-12-29

**Authors:** Feng Liu, Qinglong Guo, Guoqiang Xie, Han Zhang, Yaxi Wu, Lixia Yang

There is an error in the affiliations for the first author. Feng Liu is not affiliated with #1 and #2, but with #1 and #3: **1** Department of Postgraduate, Third Military Medical University, Chongqing, China, **3** Department of Cardiology, Kunming General Hospital of Chengdu Military Area, Yunnan, China.
